# A user-friendly graphical user interface for paw touch classification in rodent cylinder testing

**DOI:** 10.3389/fnins.2026.1803569

**Published:** 2026-08-03

**Authors:** Tyler R. Johnson, Katherine D. Zerefos, Dawn M. Taylor

**Affiliations:** 1 Department of Neurosciences, Cleveland Clinic Research, The Cleveland Clinic, Cleveland, OH, United States; 2 Department of Biomedical Engineering, Case Western Reserve University, Cleveland, OH, United States; 3 Cleveland Functional Electrical Stimulation Center of Excellence, Louis Stokes Cleveland VA Medical Center, Cleveland, OH, United States; 4 Department of Neurosciences, Case Western Reserve University, Cleveland, OH, United States

**Keywords:** 6-hydroxydopamine (6-OHDA), cylinder test, forelimb asymmetry, hemiplegia, neuroscience education, paw-touch classification, rodent video analysis software, The Ratalyzer

## Abstract

In unilateral rodent models of disease or injury, deficits are induced in only one side of the body, allowing the difference in right versus left limb usage to serve as a metric of disease/injury severity and recovery with various treatments. Unilateral rat models are commonly used for conditions such as Parkinson’s disease (via the neurotoxin 6-hydroxydopamine, 6-OHDA), stroke, and traumatic brain injury, as well as cervical spinal cord injury and peripheral nerve damage. The ‘cylinder test’ has been used for over two decades as a reliable metric of asymmetry in forelimb usage. In this test, videos are taken of the rodent in a vertical cylinder as it explores its surroundings. The experimenter then logs which forepaw was used to first touch the cylinder wall each time the animal reared up on its hind limbs to start a new wall exploration sequence. The process of manually scanning through videos and logging left/right paw touches can be very time consuming and prone to errors. To streamline this process, we made *The Ratalyzer*, an easy-to-use graphical user interface (GUI) that: (1) facilitates efficiently moving through videos to find each initial wall touch event, (2) blinds the assessor to the animal and treatment state, and (3) electronically logs the paw-touch event type and time with just a push of a button thus eliminating error-prone paper notes or manual data entry. We are making this useful software tool available to the research community as both a compiled stand-alone program and as open-source MATLAB code. This tool could be useful for both researchers and educators, as analyzing cylinder-test videos via this GUI is a great way to engage high school and undergraduate students in performing neuroscience research without the risks and costs associated with making live animals available to students for hands-on testing.

## 1 Introduction

Rodent models with unilateral forelimb sensorimotor deficits are a common way to model specific injuries or disease states where asymmetry in forelimb usage serves as a robust metric of disease severity and improvement with treatment (Schallert et al., 2000). A unilateral deficit model has the statistical advantage of each animal’s unaffected side serving as its own control, and delivering deficits to only one side of the body typically ensures the animals can still ambulate, eat, drink, and groom, thus providing a more humane option than causing deficits on both sides of the body.

Strokes in humans typically impact one side of the body more than the other. Rodent stroke models usually reproduce this asymmetry in deficits by either temporarily occluding a major artery to one side of the brain by temporarily blocking the vessel (Larpthaveesarp et al., 2021), or injecting the vasoconstrictor endothelin-1 near the artery (Abeysinghe and Roulston, 2018; Petro et al., 2016), or by injecting endothelin-1 directly into one side of the cortex (Chan et al., 2017). While Parkinson’s disease usually impacts both sides of the body in humans, common rodent models include unilateral delivery of the neurotoxin six-hydroxydopamine (6-OHDA) to just one side of the brain (Ferreira et al., 2025; Evers et al., 2024; Sung et al., 2021; Tamura et al., 2024). Other unilateral rodent injury models include spinal cord hemisections, unilateral nerve crush injuries, as well as unilateral focal brain resections or traumatic brain injuries (Chan et al., 2024; Schallert et al., 2000).

The ‘cylinder test’ for rodents was developed to quantify asymmetry in forelimb usage (Schallert et al., 2000), and it is still commonly used for this purpose today (Chan et al., 2024; Ferreira et al., 2025; Evers et al., 2024; Tamura et al., 2024). In this test, the rodent is videotaped while in a vertical cylinder. The animal’s instinct to explore the cylinder will cause it to rear up onto its hind legs and reach out with its front paws to explore and support its weight on the side walls of the cylinder. Differences in the number of times the animal uses its affected versus unaffected forepaw to first touch the side of the cylinder after rearing becomes a metric of paw-use asymmetry and a proxy for unilateral disease/injury severity (Schallert et al., 2000).

The specific parameters of the cylinder test can differ between studies, but always with the goal of quantifying asymmetry in forelimb use. Some studies only count touches when just one paw hits the wall first (Chan et al., 2024; Tassinari et al., 2020; Ferreira et al., 2025), while other studies also keep track of when both forepaws touch the wall simultaneously (Evers et al., 2024; Tamura et al., 2024). Additionally, some studies also keep track of which forepaw contacts the floor first as the rodent returns from a reared-up position to supporting its weight with all four limbs on the ground (Smit et al., 2023; Larpthaveesarp et al., 2021). The duration of the test can also vary between studies. Some researchers set a specific time limit (e.g., 5–10 min) (Chan et al., 2024; Evers et al., 2024; Smit et al., 2023) whereas others continue the test until a set number of paw touches have occurred (e.g., 20 touches) (Cuevas-Carbonell et al., 2022; Sung et al., 2021).

**Figure 1:**
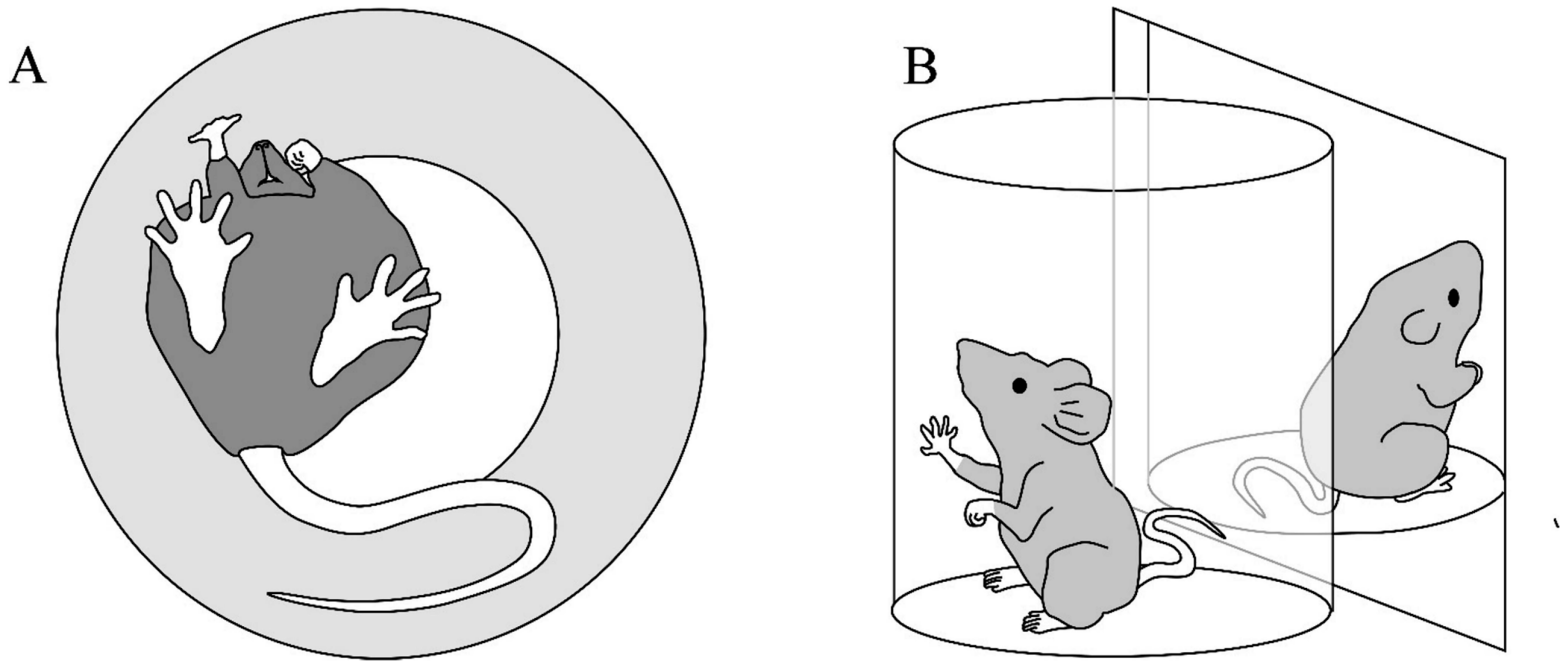
Example cylinder test camera placement options. **(A)** Rat viewed from below through a clear floor. This option allows for good detection of both wall and floor touches. **(B)** Cylinder placed next to a mirror to enable seeing the rat from multiple sides.

For accurate quantification of paw use asymmetry, rodent activity in the cylinder is virtually always recorded on video, so the experimenter can go back frame-by-frame and identify precisely when a valid wall (or floor) touch occurred and make an accurate determination of which paw touched first. Different labs use different camera placement options to be able to see paw placement on the wall regardless of the rat’s orientation. One option is to use a single camera beneath or above the animal in a cylinder with a clear bottom or top (Tassinari et al., 2020; Truong et al., 2023; Wang et al., 2024). Alternatively, the rodent can be videotaped from the side with mirrors used to ensure all sides of the animal can be seen (Tamura et al., 2024; Ferreira et al., 2025; Sun et al., 2022). Figures 1A,B illustrates two example camera setup options.

While the cylinder test has been shown to have very good inter-rater reliability (Yu et al., 2019), the process of going through videos and manually logging paw touches can be slow and time consuming. To make the process more efficient, sections of video where the rat is inactive are typically scanned at faster-than-normal speed until a potential wall/floor touch is spotted. Then, the assessor often stops the video and rewinds back past the touch segment to go through frame-by-frame to find exactly when the paw first hits the wall. Unfortunately, it is all too easy to rewind too far and accidentally re-count previously logged touches, especially if relevant touches occur close together in time. Avoiding this problem requires not only logging which paw was used, but also the timestamp in the video where the logged touch occurred.

In an attempt to make this manual video analysis process more robust and streamlined, our lab developed a user-friendly program that: (1) blinds the assessor to the animal and treatment state, (2) streamlines the process of scanning through cylinder-test videos to find paw-touch events, and (3) digitally logs those events with a simple push of a button within the GUI interface. Since our rat data were collected at two different institutions that each had their own unique recording setup, the software also needed to accommodate when each rat recording was saved as a separate video file as well as when multiple rats and/or recording days were in the same video file.

Table 1 lists the design requirements for the software. Although they are modest and focused only on making the manual data logging more efficient, blinded, and error proof, the resulting software tool has been quite useful and much appreciated by the trainees and volunteers tasked with looking through our many hundreds of rat videos.

**Table 1:** Software design requirements.

Can handle many different common digital video formats (e.g., .avi, mp4, .mov)
Can be run through MATLAB or as a stand-alone program without MATLAB.
Person assessing the paw placements can be blinded to the rat, timepoint, and treatment status via an assigned identification (ID) code
Handles recordings with multiple rats/tests in the same video file or when individual rat tests are in separate files.
Randomizes the order in which rat tests are presented for assessment
Allows one to select subsets of available rat tests to assess
Logged event labels include: ‘Left’, ‘Right’, and ‘Both’ forepaw wall touches ‘Left’, ‘Right’, and ‘Both’ forepaw floor touches An ‘Unknown’ option to use when one is uncertain which paw touched first An ‘Undo’ option in case the incorrect event label was accidentally assigned
Video controls from the GUI include: Fast-forward speed options (e.g., 2x, 3x, 5x) enabling one to quickly scan through sections of the video where the rat is inactive Normal and slower-than-normal options (e.g., 0.5x, 1x) enabling one to more carefully scan for potential paw-touch events when the rat is active A pause button that enables the following options: jump forward or back in time by 1 or 10 s move forward or backward a single frame
Logs the following results as both a MATLAB structure and a text file: Assigned animal ID codes for each cylinder test video All event labels and the times they occurred within each test. Each test’s start and stop times to allow for calculation of the *rate* of wall/floor touches as a secondary metric of overall activity.
Allows users to stop partway through a long multi-segment file and come back later to pick up where they left off

In this paper, we are making this paw-usage video analysis software tool available as a precompiled stand-alone executable program and as open-source MATLAB code. The ‘Methods’ section below walks readers through the use of the different software features, and the ‘Results’ section uses a mock data set to illustrate the software’s output and how to access and interpret the paw-touch results.

## 2 Methods

### 2.1 Program overview

The program, called *The Ratalyzer*, was initially created to streamline analysis of rat cylinder test videos at the Louis Stokes Cleveland Veterans Affairs Medical Center (Cleveland Ohio, USA), where the recording system continuously appended new test recordings to a single .avi file until reaching the maximum file size. The program was later expanded for use at the Cleveland Clinic (Cleveland Ohio, USA), where each animal test session was saved as a separate video file. Consequently, the final software accommodates both recording paradigms: multiple rats and tests per file or a single test in each file. Although no animal data from either rat study is included here, both studies under which the software was developed and refined were approved by their respective institutions’ Institutional Animal Care and Use Committees (IACUC).

While very good, general purpose, animal behavior logging software, such as BORIS (Behavioral Observation Research Interactive Software; [Friard and Gamba, 2016]) are already available to the research community, the specific needs of cylinder test data logging made it worth our while to develop our own more-specialized program that: (1) made scanning the videos and logging paw touch events very intuitive and convenient, (2) had guardrails in place to minimize erroneous entries, and, most importantly, (3) enabled blinding of the video assessors to the animal cohort and treatment state. While BORIS enables customized event marking using assigned keyboard ‘hotkeys’ or touchscreen menus, we wanted the convenience of controlling both the video playback and event logging using mouse-click activated GUI buttons that were logically-organized directly under the video image. This ensured the person logging the paw-touch events did not have to move their hand between mouse and keyboard (or touch screen) or move their eyes between screen and keyboard. Importantly, our code is set up to prevent double counting of events by preventing re-assessment of video sections that have already been logged. Additionally, the Ratalyzer code enables us to blind the assessor to the cohort and treatment state by (1) only allowing the assessor to see limited sections of videos where identifying information about the animal or treatment state was not in view, by (2) facilitating the assignment of coded animal IDs, and by (3) randomizing the order in which the video segments are presented for assessment to eliminate temporal cues that could reveal treatment stage. To our knowledge, there are no commercial software packages available at this time that meet all these criteria. Although many companies working in the rodent behavior software space are willing to make custom software tools for a price, this option is typically beyond the budget of many research labs. Here we have made this useful task-focused software tool freely available as a stand-alone program and as editable source code to facilitate its use by any research group who could benefit from it.

Here we describe the workflow and blinding process for using the Ratalyzer tool under three main recording scenarios to accommodate different lab’s recording constrains and animal/file labeling systems: (1) when multiple recorded tests are in the same video file and animal identifiers are briefly placed in camera view at the start of each new animal test, (2) when each file contains only one animal test but still includes identifying information or excess video that needs to be trimmed, and (3) when each video file only contains one rat test and no identifiers or excess video.

The analysis process consists of two main stages—an unblinded ‘video segmentation stage’ and a blinded ‘touch classification stage’.

1) *Video segmentation* (unblinded)—For videos with multiple tests/rats per video, this stage is where the experimenter goes through the videos to identify the start and stop times of each individual rat’s cylinder test and manually assigns a corresponding animal code to each video segment. This step is typically *not* blinded as the experimenter needs to know which rat test file is being associated with each blinding code. For recordings where the animal’s identifying information was logged by placing an identification card at the start of the video itself, this step is where the analysis start and stop times are set to exclude any identifying sections of video so the person classifying the touches in the next step cannot see the true identifiers. For videos that only contain one individual rat’s cylinder test with no identifiers, this segmentation step can be skipped and coded video file names can be used to ensure blinding in the next step. More details on blinding are included below.
2) *Touch classification* (blinded)—In this stage, the blinded assessor goes through the rat test videos to mark when various paw-touch events occurred. The randomization option here ensures the files associated with the selected rat ID codes are presented in random order across rats as well as across different timepoints for each rat.

**Figure 2:**
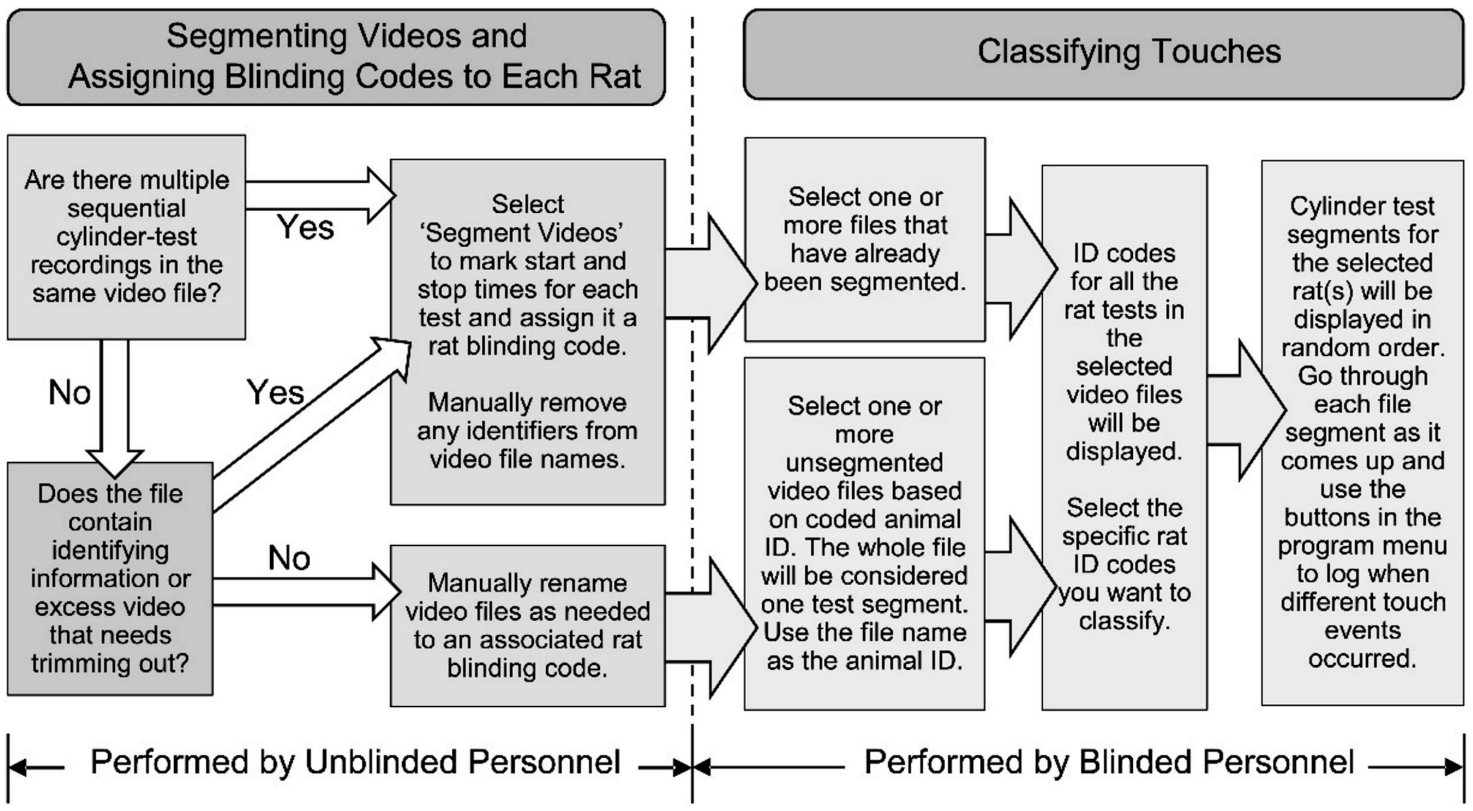
Flowchart illustrating The Ratalyzer’s workflow for different recording scenarios.

Figure 2 shows the workflow through these two processing stages for each of the three recording scenarios accommodated by this software.

### 2.2 Program setup and organization

The program folder, installation instructions, executable, and associated files can be downloaded from Zenodo at https://doi.org/10.5281/zenodo.15750960. The program was developed in MATLAB App Designer (version 2019a) and can be executed either directly within MATLAB by running file “Ratalyzer.mlapp” or as a stand-alone application by clicking on “Ratalyzer.exe.” For users running the stand-alone executable version, MATLAB Runtime Library version 9.6 is required and is freely available from MathWorks.^1^ Due to the program’s reliance on Windows-specific directories, the software is currently compatible only with Windows operating systems. However, it should be easily adapted to other platforms via the open-source code we have made available on GitHub.

The required codec for your video file format must be installed on your system and must be supported by MATLAB. Many MATLAB-compatible codec and codec packages can be found here: https://codecguide.com/download_kl.htm. Specifically, the software supports common video formats including .mp4, .avi, .mov, .mkv, .mpg, and .wmv, and dynamically adjusts to any frame rate natively supported by the host system’s codecs. To optimize memory requirements and ensure stable performance on standard laboratory computers, the software’s architecture utilizes MATLAB’s VideoReader object to read media sequentially frame-by-frame rather than loading entire video files into active memory. This design keeps the memory footprint minimal, allowing the program to easily handle extremely large, multi-hour recordings without performance degradation. Additionally, the application features built-in exception handling for media initialization so that if a corrupted file or unsupported codec is encountered, the program safely catches the error and alerts the user rather than experiencing a hard crash.

Importantly, the software does not alter the original video files in any way, thus maintaining the integrity of the original source data files. Instead, the segmentation process simply defines the start and stop times of the data segments to be analyzed, and only those sections of the video are accessible to the assessor during the touch classification phase. This process is more computationally efficient than saving out new trimmed video files for each segment, which would be the alternative if animal/treatment identifiers are in the video and one wanted to blind the assessor to the animal cohort and treatment state without using a software tool like this.

To use the program, the video files to be analyzed should first be placed in the “Videos” subfolder within the main Ratalyzer folder containing the executable files. When starting the Ratalyzer application, the program GUI will open in initial startup mode with a list of all video files found in the “Videos” subfolder. To ensure blinding of the assessor to treatment conditions, the videos to be analyzed should be given names that do not give away information about the rat cohort, treatment states, or time periods being assessed.

**Figure 3:**
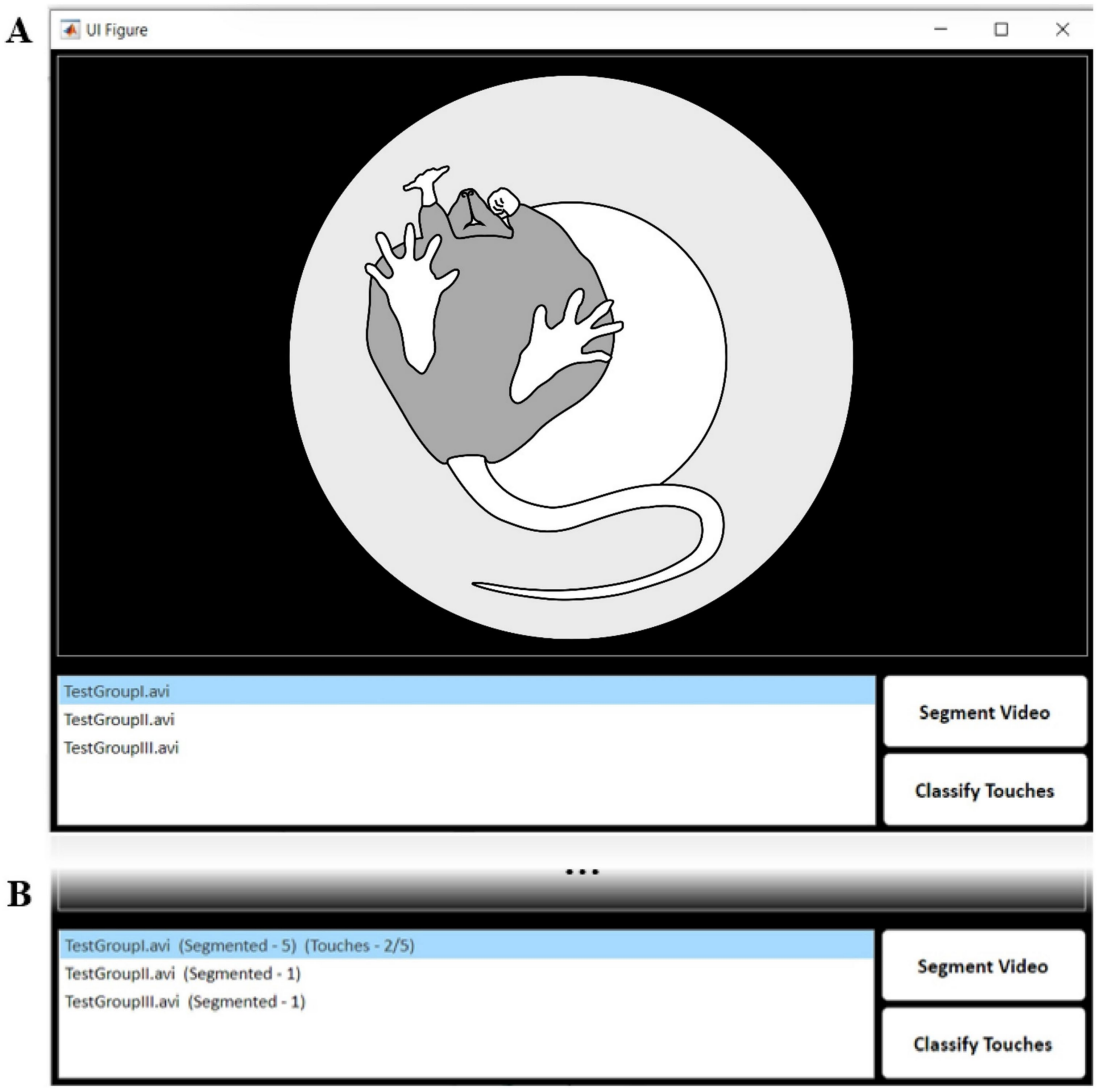
The Ratalyzer home screen containing both the video playback screen on top and processing controls at the bottom of the same window. The controls at the bottom of part **(A)** show three videos are available for processing and that none of them have been segmented or had touches classified yet. Part **(B)** shows the video list after all files have been segmented, and one file has had touches classified for two of the five test segments.

In this initial home screen, there are two options that can be applied to the different selected video files: (1) ‘Segment Video’ which can only be applied to one video file at a time, or (2) ‘Classify Touches’ which can be applied to single files or groups of selected files (Figure 3A). After a video has been segmented, the video list on the home screen is updated with the number of identified segments in that file. Similarly, once touch classification has started, the number of segments in which touches have been identified will also be listed in the home screen as shown in Figure 3B where ‘(Touches—2/5)’ indicates that two of the five segments in that file have already had touches classified.

### 2.3 Segmenting and de-identifying videos

A ‘Segment Video’ step must be performed first for files that have multiple cylinder tests recorded in the same file or that have sections of the video where the rat’s true identifiers or treatment conditions are visible and need to be cut out of the video to keep the individuals doing the touch classification blinded to the rat’s status.

**Figure 4:**
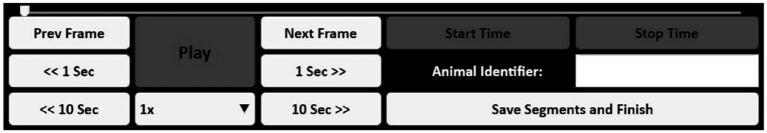
File segmentation menu. An animal identifier code should be typed into the field before defining start and stop times. The pulldown menu under the ‘play’ button allows one to set playback speed to 0.5, 1, 2, 3, or 5 times normal speed.

The segmentation step is where the start and stop times of each individual cylinder test are assigned (Figure 4). The Segmentation menu will not allow one to define start and stop times for a segment until an animal code is manually entered into the ‘Animal Identifier’ box. The specific rat, timepoint or treatment state associated with this code should not be recognizable to the future blinded assessor to ensure the animal’s treatment state cannot be figured out from these ID codes. As one of the software’s guardrails against errors, the program will flash a warning and not allow for start and stop times from neighboring segments to overlap or for stop times to precede start times.

Once all identified cylinder test segments in a given video file have had animal ID codes and individual test start/stop times defined, clicking finish will return the program to the home menu. Any file with defined segments will now have the number of segments displayed after the file name (See Figure 3B as an example). Selecting a file that already has segments defined and clicking ‘Segment Video’ again will bring up a menu option of either ‘overwriting’ or ‘appending’ to existing segments or ‘cancelling’ the request.

### 2.4 Classifying Touches

The ‘Classify Touches’ mode is where the blinded assessor scans through the various cylinder test segments and marks when right or left paw touches occurred. If a given file does not yet have any associated segments and is selected for touch classification, the program will ask for an associated animal code and treat the entire file as a single segment. In this case, the file names should have already been assigned the animals blinding code either at the data collection phase or by unblinded personnel after data collection.

**Figure 5:**
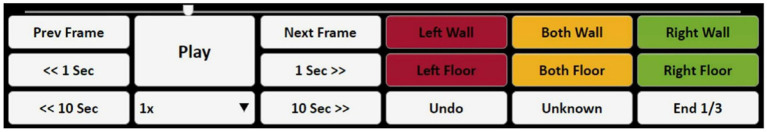
Touch classification menu: Playback controls are the same as in the segmentation menu. Additional buttons specify the seven touch options (e.g., combinations of ‘Left’ vs. ‘Right’ vs. ‘Both’ paws and ‘Wall’ vs. ‘Floor’ placement plus ‘Unknown’). The ‘Undo’ button erases the most recently selected touch option. ‘End x/x’ terminates the current segment and either moves to the next available segment or returns to the home screen if all segments have been completed.

Figure 5 shows the menu available in the ‘Classify Touches’ mode. As rat videos often have long sections of time where the rat is just resting or grooming and is not exploring the cage, we have found the most practical way to use this tool is to play the video at 2x, 3x, or 5x speed to quickly scan through these uneventful sections and then slow the video down to 1x or 0.5x speed once the rat starts exploring the cylinder. Then, when a potential paw touch is spotted in this slower mode, pause the video and use the jump back 1 (or 10) second(s) button to rewind to just before the event occurred. Then one can advance the video frame-by-frame to identify the exact time of first contact and determine which paw touched first.

During playback, navigation is restricted to within the active segment to prevent accidentally jumping between different cylinder test segments. To prevent touches from being double counted, the program also does not allow users to go back past the last defined touch. Therefore, it is important to classify each touch in order. If one is uncertain which paw touched first, selecting ‘Unknown’ marks the event, which can then be reviewed *post hoc* using the video time code in the results file to get a second opinion if needed. One can also choose not to log unclear paw touches thus excluding them from the analysis. However, logging them as ‘Unknown’ allows for more accurate calculations of the *rate* of paw touches as an additional useful metric of overall activity levels.

When one has marked all identified touches, and the end of the segment has been reached, the video will not advance any further. Selecting the ‘End x/x’ button will either advance to the next segment, if there are still segments to classify, or else it will terminate the classify-touch mode and return to the home menu. The entire program can be closed simply by clicking on the X in the upper right corner of the window.

**Figure 6:**
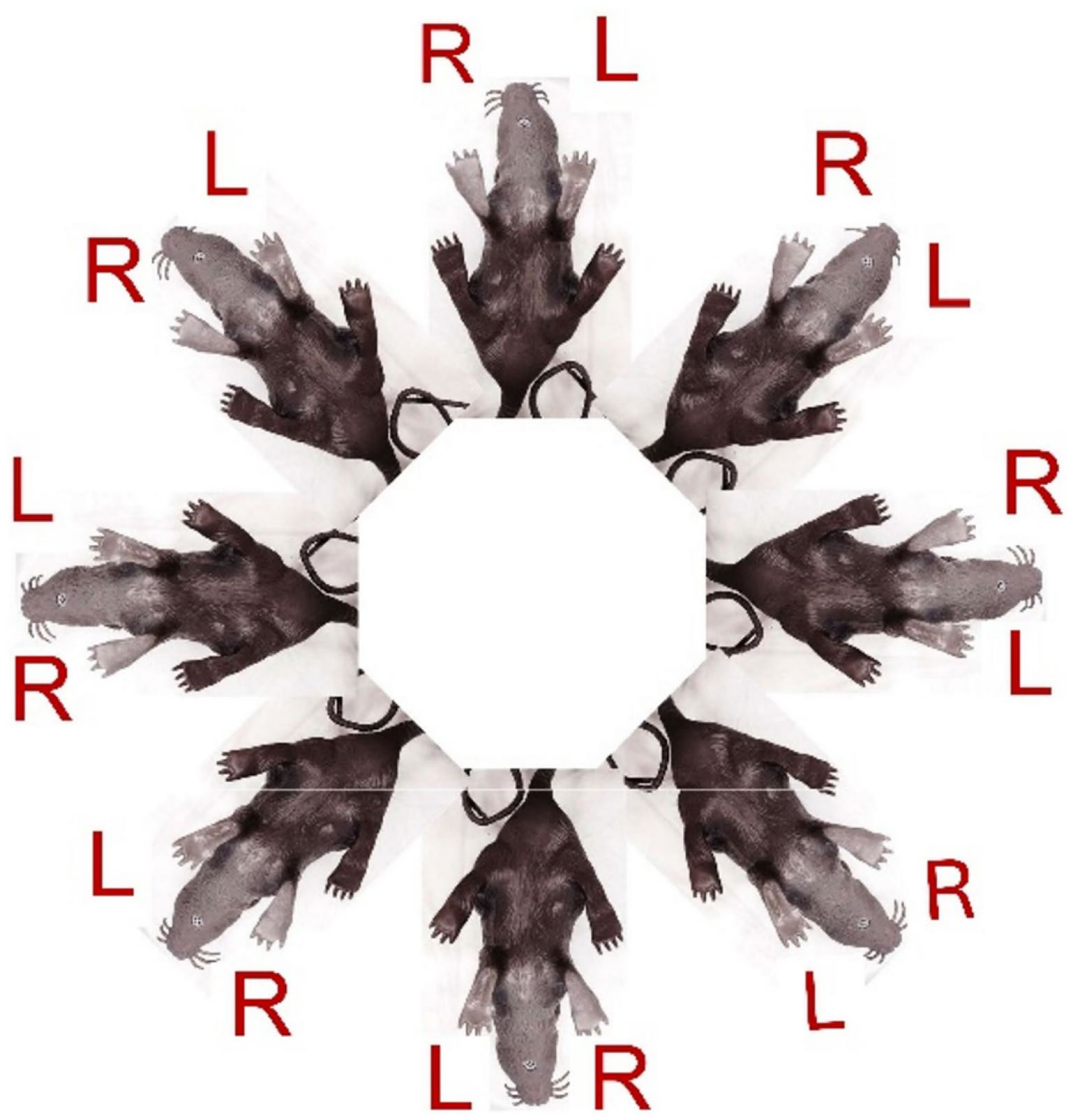
Example left/right paw reference guide used to enable assessors to quickly discriminate left vs. right paw usage when the rat is in any orientation.

One might think determining if a paw touch was with the right or left paw would be easy. However, errors can be common at this step, especially when the rats are in a confusing orientation and/or the paw touch must be identified in the mirror image. To minimize classification errors, we have made several reference guides for different camera setups to help assessors quickly identify which paw is which when the rat is in different orientations. Figure 6 illustrates an example reference guide for the recording setup illustrated in Figure 1A. Full-sized PDFs of this and additional reference guides for other camera setups are available for printing and can be found in the main Ratalyzer folder on GitHub.

### 2.5 Blinding and randomization

The software is designed to blind the person doing touch classification using both coded rat identifiers unknown to the assessor and by randomizing presentation order to avoid conveying temporal information about an animal’s treatment stage. Blinding to the particular rat cohort and treatment/disease state is critical for preventing unconscious (or intentional) bias as the process of determining which paw touched the wall first is not always clear cut, especially if the video quality is poor, the left and right paws touch in close succession, or a paw is held near the wall without actually making contact. Figure 2 shows which parts of the workflow should be performed by unblinded personnel and which steps should be performed by blinded personnel for each of the three workflow options.

The choice of naming conventions for the video files and animal ID codes is up to the individual lab as is how one stores the information linking these file names and manually assigned ID codes to the actual animal IDs and treatment state information. Historically this information would have been stored in a physical lab notebook kept inaccessible to any blinded assessors. However, one of the many available ‘electronic lab notebook’ programs with configurable permissions settings may be a safer option due to the automatic backing up of these key data records.

A unique ID code should be assigned to each rat and could even be assigned to each individual test if needed. However, assigning one code per rat has the added advantage that touch classification can be done in batches for individual rats or subsets of rats. These options are useful if certain rats need to be analyzed right away or if a rat must be dropped from the study part way through and there is no longer a need to continue analyzing its data.

**Figure 7:**
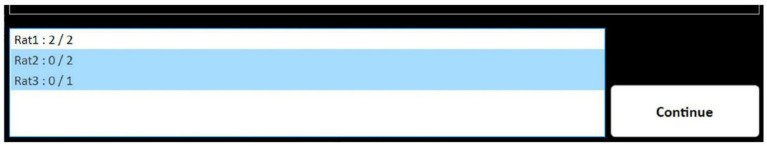
Rat selection menu for touch classification. This menu allows assessors to select a specific rat or subset of rats for touch classification.

Figure 7 shows an example animal selection window that is displayed once one or more files are chosen for touch classification. The software displays code IDs for all the rats with test segments in the selected video files as well as the touch-classification completion status of those segments. Clicking on specific rats will allow the assessor to only classify touches for the chosen rats which can then be presented for touch classification in a random order. The application uses MATLAB’s ‘randperm’ function to shuffle the presentation order of the selected video files and segments. The resulting randomized sequence is not reproducible, as the state of the random number generator is neither set to a static value prior to classification nor captured and saved alongside the exported data.

Since the segmentation phase and the touch classification phase should be done by different people, often these tasks are performed on different computers. To allow for this scenario, one would simply install the complete Ratalyzer folder on both computers. Once the unblinded person is done segmenting the files, the ‘Videos’ folder and ‘VideoData.mat’ file can simply be transferred to the touch classification computer for blinded assessment. Keep in mind that the raw transferred videos may still contain identifiers although the Ratalyzer program will not display those sections of the video during the touch classification process. If one anticipates an assessor may *maliciously* go directly to the video file outside of the Ratalyzer program to try to look up the hidden true animal ID, the following steps could be taken outside of the Ratalyzer program to prevent malicious unblinding: (1) avoid recording any animal identifiers on screen in the first place by either recording each animal test in a separate file or by using timestamps to track different animal tests if your recording system is set up for recording multiple animals per file, or (2) trim out identifiers and save individual files by coded animal ID using any video editing program.

## 3 Results

**Figure 8:**
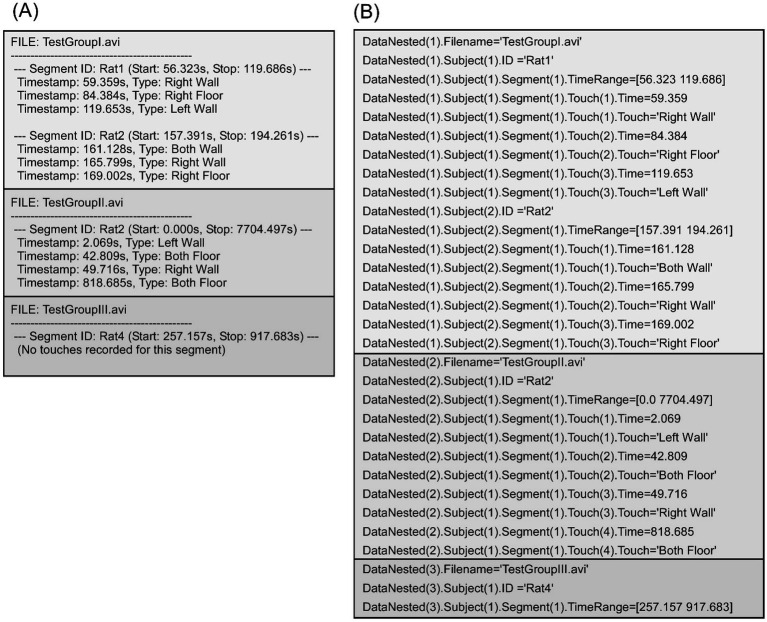
Format of ‘UserData’ results files: An abbreviated example output shows data formatting for the .txt file **(A)** and the data structure fields in the .mat file **(B)**.

Upon termination of the program, all results are written out to both a text file (UserData.txt) and a MATLAB file (UserData.mat) which are both saved in the main Ratalyzer folder. Figure 8A shows an example text file output, and Figure 8B shows the same data output in its MATLAB file structure. For the text file, individual video file results are listed in alphabetical order while segments and touches are listed in sequential order within each video file section. The MATLAB file contains one variable ‘DataNested’ which is a 1xN structure array where N is the number of videos. The DataNested schema is organized as a nested structure array descending from File → Subject → Segment → Touch. Table 2 summarizes the ‘DataNested’ Structure contents. Because the data is already grouped by subject and segment, users can easily extract key metrics using MATLAB scripts customized to one’s needs. Example MATLAB scripts are included in the downloaded folder that shows how to extract summary results from this MATLAB data structure.

**Table 2:** Matlab nested data structure fields and contents.

Matlab structure fields	Contents
DataNested(i).Filename	*String representing the video file name*
DataNested(i).Subject(j).ID	*String of the blinded animal code*
DataNested(i).Subject(j).Segment(k).TimeRange	*A 1 × 2 double array containing the start and stop times of the specific testing segment*
DataNested(i).Subject(j).Segment(k).Touch(m).Time	*Double representing the exact timestamp of the paw touch*
DataNested(i).Subject(j).Segment(k).Touch(m).Touch	*String specifying the event classification (e.g., ‘Left Wall’, ‘Right Floor’, ‘Both Wall’, etc.)*

The program is designed to allow assessors to stop analysis between test segments and pick up where they left off at a later time. An additional file, VideoData.mat, is written out upon program termination and is used by the program to save progress information for reloading the next time the program is opened. This file needs to remain in place to ensure the program correctly recognizes what processing steps have already been completed. Once all assessments have been performed on the videos in the Videos folder, those videos and associated results files can be moved and stored elsewhere and new videos added for assessment.

## 4 Discussion

Cylinder testing is used to quantify asymmetric rodent forepaw usage as a way of assessing the efficacy of various treatments in unilateral rodent models of injury or disease (Schallert et al., 2000). However, the process of manually assessing rat videos to identify when left or right forepaw-touch events occurred can be very time consuming and prone to errors. The *Ratalyzer* software utility was developed with the goal of make the process of scanning through videos and logging paw touch events much faster, easier, and more reliable. While the software still relies on human judgement to spot and classify each paw touch event, the software video control panel is designed to facilitate homing in on paw touch events and then record with a single button push both the event time and the assessor’s decision on which paw touched first. This software eliminates cumbersome paper notes or manually typing data into an electronic log file after each identified event, which are both susceptible to human error.

In our own lab, the *Ratalyzer* software interface was iteratively revised over time based on feedback from the assessors regarding what would make the event logging process easier and more efficient. Anecdotally, the added features the assessors reported most appreciating were the ability to log events with on-screen menu buttons instead of keystrokes and the on-screen video navigation buttons that facilitated (i) rapid scanning through low-activity video segments, (ii) stopping and rewinding back past a potential touch event once one was spotted, and (iii) stepping frame-by-frame through an event with a click of a button to be able to carefully assess the image at the exact time of contact. The specific time savings and reduction in data logging errors one can expect from this software will depend on many factors including the experience of the assessor, the quality of the video, the behavior of the animal, and the original data logging methods used. However, studies have shown that spatially organized GUI buttons are one of the most efficient data logging options (Fernandez and Husser, 2013). Here we have organized the buttons with left/right and wall/floor options spatially organized to maximize intuitive use.

Safety features built into the software are designed to reduce the chances that the same segment of video will get accidentally double counted as two different cylinder tests. Similarly, the software is designed to prevent a single paw-touch event from being double counted as two separate touches. Importantly, the software facilitates the blinding of the assessor doing the touch classification to the rat ID, timepoint, or treatment status thus reducing the potential for unintentional (or intentional) bias in determining forepaw usage.

Here we have made the software tool freely available to the research community as both a stand-alone executable that can be run directly or an option that is run through MATLAB. Importantly, the source code is provided to encourage further development and customization by future users. Further development could include developing Mac and Linux compatible versions. The Ratalyzer utility could also be incorporated into a larger data process stream for large throughput studies that, for example, automatically rename individually-recorded animal files with coded IDs as well as automatically unblind and calculate summary metrics from the coded output files.

To further facilitate accurate classification of left versus right forepaw use, we have also developed and made available several handy reference guides illustrating a rat in various orientations and camera views with the left versus right paw clearly marked. These guides can be enormously helpful to prevent misidentification of the left versus right limb, especially when the paw is only visible in the mirror or the rat is rotated within the camera view.

Although we developed the program for use in our small lab studies, the features of the software make it amenable to large throughput studies. Specifically, the program’s frame-by-frame reading enables large video files to be processed without needing to load the whole file in memory, and any number of files or animal segments can be selected for batch processing as the file/segment list is not limited in size. Although the manual paw classification process is inherently labor intensive, the process gets faster as assessors get more experience in rapidly scanning through videos to home in on potential paw-touch events and more experience discerning how the paw shape changes as it makes contact with the wall or floor viewed from different angles.

One of the largest factors in determining touch classification speed is the quality of the video image. Recording videos at a higher pixel resolution and higher frame rate can make discerning paw-touch events more accurate but with the trade-off of increasing the file size as well as requiring more expensive equipment. This software tool is designed to work with a wide range of video formats and frame rates. One way to improve video quality without upgrading one’s equipment is to use stronger lighting, which has the advantage of both improving overall visibility and increasing the range of depths where the wall touches will be in focus due to the smaller lens aperture needed. However, since rodents are nocturnal animals, many labs, including ours, have their rodents on reverse light cycles so the animals will be more active in the dark for behavioral testing during normal work hours. Keeping light levels low during testing may be preferable for maintaining the animal’s circadian rhythm, encouraging cylinder exploration, and preventing drowsiness and inactivity during cylinder testing. In our own dim-light testing setup, in spite of the lower video quality, the images were clear enough that the assessors only labeled putative paw touches as ‘unknown’ about 6 % of the time as an indication that they were unable to determine with confidence if a touch occurred or which paw touched first.

Even with the highest resolution cameras in the best lighting conditions, there will inevitably be cases where paw-touch events will be missed due to other body parts blocking the camera’s view. Given how the equipment, lighting, and assessor training can all impact the accuracy with which paw touch events are classified, it is important to keep these factors as consistent as possible across the animal cohorts being compared.

In our own lab, we compared Ratalyzer classification results from two experienced assessors to their previous manual assessments of three rats performing ~90 paw touch events. Notably, classification results were more consistent between the methods used than between the two assessors themselves. Classification results for the two experienced assessors were 90 and 96% consistent between the Ratalyzer and manual assessment methods. However, classification results were only 85% consistent between the two assessors for both the manual and Ratalyzer methods. After reviewing the files more closely, common classification differences between assessors resulted from different criteria being used to decide if the two paws touched close enough together in time to be classified as ‘both’ versus a ‘right’ or ‘left’ touch event. Additional differences were due to one assessor more frequently using the ‘unknown’ classification when the visibility was poor whereas the other assessor was more willing to make an educated guess. The assessor that used the ‘unknown’ classification more often was also the one with the highest (96%) consistency between the manual and Ratalyzer results, compared to the 90% consistency across methods for the assessor that tried to make a classification even when paw visibility was poor. When removing the events with at least one classification listed as ‘unknown’, classification results improved to 95 and 97% consistent between the Ratalyzer and manual assessment methods for the two assessors, and consistency between the two assessors increased to 91% for both methods. These examples highlight the need for good communication and consensus between assessors on the criteria to be used when making these inherently subjective classification decisions.

Advances in markerless pose estimation software, such as DeepLabCut (Mathis et al., 2018), have shown remarkable progress in capturing rodent paw biomechanics in conditions where the animal is in a consistent location relative to the camera over the course of many trials (Bova et al., 2019). Pose estimation has also gotten very good at tracking rodent in-cage ambulation (Lauer et al., 2022). However, automating the process of extracting accurate paw-wall interactions during cylinder testing via pose estimation is still particularly challenging because the animal is free to move anywhere in the cylinder, and wall touches can occur at any wall location and orientation relative to the camera view. Nevertheless, machine learning and pose-estimation tools could potentially be used to further improve results obtained with the *Ratalyzer* tool by displaying estimated bone and joint locations over the video prior to running it through the *Ratalyzer* software for manual touch classification.

Conversely, the *Ratalyzer* tool could also be adapted to further the development of pose estimation and artificial intelligence (AI) for detection of wall-paw interactions: (1) by allowing the assessor to quickly spot and log inaccurate paw pose estimates that need correcting, and (2) by contributing to the very large data set of correctly-labeled and validated pose-estimates of wall-touch interactions that will be needed to train effective AI tools to replace the manual cylinder-test assessment process.

Even as better AI tools are developed in the future, the process of manual paw-touch identification of cylinder test videos will always be a great research learning experience for undergraduate neuroscience students. The video-based paw-touch classification process allows large numbers of students to participate in the animal research process without the costs and risks that come with live animal experiments performed by students. The cylinder test and *Ratalyzer* software tool can be a great way to teach students about the sources of error and variability in experimental data, the importance of blinding the person measuring results to the treatment conditions, and experimental design considerations such as the need for control groups and how to appropriately power a study.

By making the *Ratalyzer* software freely available, this tool can be used ‘as is’ and/or further improved by the research community to aid in the evaluation of different treatments in unilateral rodent models of injury or disease, potentially facilitating preclinical research on neurological conditions. The open-source software could also be adapted in the future for use in a wider range of behavioral analysis applications, such as studies on natural limb use laterality, neurodevelopment, motor asymmetry, and cognitive behavioral testing. In some cases, the adaptation process could be as simple as changing the paw placement event label buttons to whatever parameters are relevant to the particular study while making use of the existing core workflow for video segmentation, randomized presentation, and blinded timestamped event logging.

## Data Availability

Publicly available datasets were analyzed in this study. The Ratalyzer program as well as all code used to make the program can be found at: https://doi.org/10.5281/zenodo.15750960.

## Footnotes

1
https://www.mathworks.com/products/compiler/matlab-runtime.html
